# Osteoblast behaviours on nanorod hydroxyapatite-grafted glass surfaces

**DOI:** 10.1186/s40824-019-0178-6

**Published:** 2019-12-21

**Authors:** So Jung Park, Kailash Chandra Gupta, Hun Kim, Sukyoung Kim, Inn-Kyu Kang

**Affiliations:** 1 Department of Polymer Science and Engineering, Kyungpook National University, Daegu, 702-701 South Korea; 2 Department of Chemistry, Polymer Research Laboratory, I. I. T. Roorkee, Roorkee, 247 667 India; 3 Jeil Medical Corporation, Seoul, 08378 South Korea; 4 School of Materials Science and Engineering, Yeungnam University, Gyeongbuk, 712-749 South Korea

**Keywords:** Glass, Nanorod hydroxyapatite (nHA), Surface analysis, Bioactive and MC3T3E1 cells

## Abstract

**Background:**

The goal of this study is to obtain basic information to improve the bone adhesion of silica components, which are used as the main ingredient in glass ionomer cement (GIC). To achieve this, nanorod hydroxyapatite (nHA) was grafted to the surface of silica cover glass. Surface analysis confirmed nHA was joined to the glass surface and biocompatibility with osteoblasts was investigated.

**Results:**

The grafting of nHA on the surface of slide cover glass (Glass) was confirmed by X-ray photoelectron spectroscopy (XPS) and contact angle (θ) measurement. MC3T3-E1 cells were more stretched out on the nHA-grafted cover glass (Glass-nHA) in comparison to the Glass. In addition, the Glass-nHA was more bioactive in supporting the proliferation of MC3T3-E1 cells in comparison to cells seeded on the Glass.

**Conclusion:**

The Glass-nHA was to be highly bioactive and this might be useful information for property modification of GIC.

## Introduction

Glass ionomer cement (GIC) is the most popular restorative materials and has been used for last 3 decades due to its biocompatibility with structures and properties of teeth constituents [1]. The hydroxyapatite (HA) is found to be highly beneficial in the field of restorative dentistry due to its intrinsic radiopaque response and other properties [2, 3]. Currently the application of nanoscale biomaterials is found to be potentially more useful in dentistry and becoming more acceptable due to having better properties in terms of strength, polishability and aesthetic value in comparison to commercially used modifiers [4, 5]. The recent advancements in synthesis of HA [6] in different size and forms have made it possible to use HA as biocompatible analogue filler to natural teeth materials. Besides, HA has shown excellent biological activities and played vital role in orthopedics applications due to its favorable osteoconductive and bioactive properties.

The application of HA has shown a significant improvements in mechanical properties such as compressive strength, tensile strength [2] and flexural strength [7], and also in bonding with teeth dentin and providing fluoride-releasing properties [8]. To increase the efficiency of HA in controlling the mechanical properties of GIC, the one pot synthesized silica-hydroxyapatite (Si-HA) [9] has been recently reported [10, 11]. The secondary caries are considered responsible for frequent application of restorative GIC [12]. Therefore, applying of hydroxyapatite nanoparticles in GIC could be a solution for dental restoration and in enabling dentin to combat secondary caries. The HA is found to be highly bioactive materials and constitute a major part of dental enamel and bone tissue. Because of its structural similarity to human teeth and skeletal system, its application in restorative materials is proved to be potentially useful in various studies dealing with dental materials [13]. The application of nano-sized paticles in GIC led to their wider distribution and better reinforcement in GICs.

To understand the reaction of nanorod hydroxyapatite (nHA) with silica surfaces, this study has been carried out using L-glutamic acid-grafted nHA and slide cover glass (Glass) to prepare the nHA-grafted cover glass (Glass-nHA). The Glass-nHA was characterized by scanning electron microscopy (SEM), Fourier transform infrared (FT-IR) and X-ray photoelectron spectroscopy (XPS). Osteoblast behaviors on the Glass-nHA were also evaluated by SEM, water soluble tetrazolium salts (WST-1) assay and live/dead assay.

## Experimental

### Materials

3-Aminopropyltriethoxysilane (APTES) as silane coupling agent, N-(3-dimethylaminopropyl)-N′-ethylcarbodiimide hydrochloride (EDC), N-hydroxysuccinimide (NHS) and paraformaldehyde were purchased from Sigma Aldrich Chemical Company, USA and used as received. L-Glutamic acid and 25% glutaraldehyde solution was purchased from Junsei, Japan. Premix WST-1 solution was purchased from Takara, Japan. Calcein-AM and ethidium homodimer III staining solution were purchased from Biotium, USA. Dulbecco’s phosphate buffered saline (DPBS) solution, dulbecco’s modified eagle medium (DMEM) were purchased from Gibco. The mouse pre-osteoblast cells (MC3T3-E1) were purchased from Korea cells bank (Seoul, South Korea). The MC3T3-E1 cells were cultured in DMEM supplemented with 10% fetal bovine serum (FBS; Gibco), 1.0% penicillin G-streptomycin at 37 °C under 5% CO_2_ atmosphere. The culture medium was changed in every 3 days. The nHA were synthesized as per details as given in our previous communication [14]. Cover glass (borosilicate glass type D 263® M) with a thickness of 0.13 to 0.16 mm and a radius with 15 mm was procured from Paul Marienfeld GmbH & Co. KG, Lauda-Königshofen, Germany.

### Surface modification of glass by APTES

**Fig. 1:**
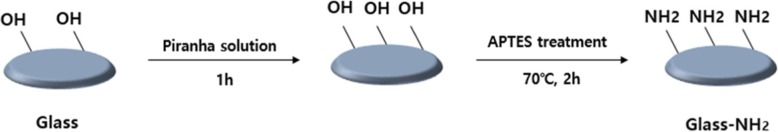
Introduction of primary amine to the surface of the Glass

The acetone-cleaned Glass was treated with piranha solution (1:3, H_2_O_2_:H_2_SO_4_) for approximately 1 h before use to produce hydroxyl groups on the surface (Fig. 1). After removing the excess amount of unused piranha solution, the Glass was washed three times (5 min each) with deionized water. After the treatment, the Glass was kept in 2 wt% solution of APTES in 95% ethyl alcohol for 2 h at 70 °C (Fig. 1). Finally, the Glass was rinsed first time with ethanol and then two times with deionized water. The Glass was ultrasonicated for 30 s to remove physically adsorbed APTES and dried in a vacuum chamber for 4 h at 70 °C. To confirm the surface modification of the Glass by APTES, the contact angle measurements of surface-modified Glass was carried out by sessile drop method using a ráme-hart goniometer (Mountain Lakes, NJ, USA) and XPS spectra were recorded using ESCALAB MK II X-ray photoelectron spectrometer (VG Scientific Ltd., England).

### Grafting of nHA to APTES-grafted glass

**Fig. 2:**
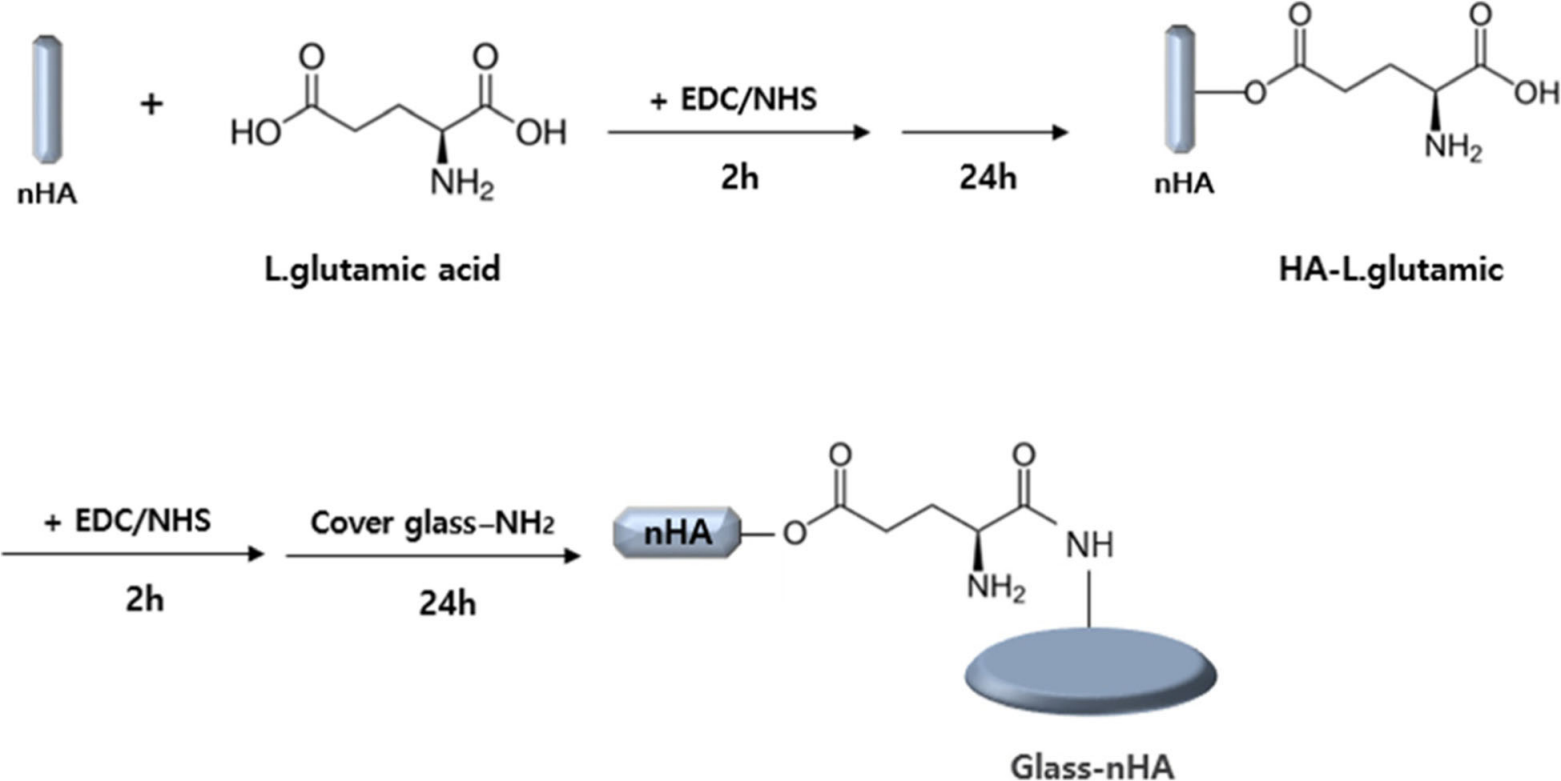
Surface modification of the Glass with L-glutamic acid-grafted nHA

To graft the nHA on the surface of the APTES-grafted Glass, nHA nanoparticles were synthesized through precipitation method as per our previous reports [14]. Dihydrogen ammonium phosphate ((NH_4_)H_2_PO_4_) and calcium nitrate (Ca (NO_3_)_2_4H_2_O) were used as each phosphorus and calcium precursors and ammonia water was used to adjust pH 10.4 of reaction solution. The formation of nHA was confirmed by transmission electron microscope (TEM). To increase the reactivity of nHA with the APTES-grafted Glass, the surface of nHA was grafted with L-glutamic acid as described in our previous communications [14, 15]. Briefly, the terminals carboxylic acid groups (−COOH) of L-glutamic acid were activated by taking 0.55 g of L-glutamic acid in 200 mL mixture of water-soluble 1-ethyl-3-(3-dimethylaminopropyl) carbodiimide hydrochloride (0.5 g, 0.25 wt%) and *N*-hydroxysuccinimide (0.5 g, 0.25 wt%) under constant stirring. After activation time for 2 h, 0.5 g of nHA was added, and the solution was allowed to stir continuously for 24 h (Fig. 2). Finally, a solution containing L-glutamic acid-grafted nHA particles were centrifuged and freeze dried after washing three times with deionized water. Surface modification of nHA was confirmed by taking FT-IR (Galaxy 7020A; Mattson, Fremont, CA, USA). To immobilize the L-glutamic acid-grafted nHA on APTES-grafted Glass, 0.5 g of dried and purified L-glutamic acid-grafted nHA particles were added to 100 mL mixture of EDC (0.5 g, 0.25 wt%) and NHS (0.5 g, 0.25 wt%) under constant stirring. After 2 h, the APTES-grafted Glass was immersed in the solution and kept for 24 h (Fig. 2). Finally, the surface-modified Glass was taken out and rinsed three times with deionized water and ultrasonicated for 30s to remove the unreacted activating agents and other impurities. After drying, the Glass-nHA was subjected to the measurement of water contact angle (θ). The measured contact angle (θ) was reported as an average of three measurements. The surface morphology of Glass-nHA was analyzed by recording FE-SEM micrographs (400-Hitachi, Tokyo, Japan). To record the SEM micrographs, a piece of the Glass-nHA was fixed to SEM holder using double adhesive carbon tape and then sputter-coated with platinum. The platinum-coated sample was then examined by FE-SEM under high vacuum. To confirm the grafting of nHA on the Glass and to determine the weight percent of elements present in the Glass-nHA, the Glass and the Glass-nHA were recorded using XPS. To confirm the anchoring of nHA on the Glass, energy dispersive spectroscopy (EDS) was also recorded using FE-SEM equipped with energy dispersive analyzer.

### Cellular behavior on glass and glass-nHA

To determine the effect of nHA grafting on the cell behaviors of the Glass, the adhesion and proliferation of MC3T3-E1 (osteoblast cells) were determined using cell based assay protocols.

### Cell adhesion

To assess the attachment of cells to the Glass and the Glass-nHA, the MC3T3-E1 cells (4 × 10^4^ cells/mL) were seeded, respectively, on each glass. The cell seeded glasses were incubated at 37 °C, 5% CO_2_ in humidified atmospheres for 1 and 2 days in presence of DMEM medium. After culturing, the supernatant was removed and each substrate was washed three times with PBS and fixed with a 2.5% glutaraldehyde solution for 1 h. The samples were then dehydrated through a graded ethanol series, dried in a critical point drier and sputter-coated with platinum before recording SEM micrographs.

### Cell proliferation

Various methods such as MTT, XTT and MTS are used for determining of cell viability and proliferation based on colorimetric detection of absorbance corresponding to the number of viable cells [16, 17]. However, in these methods, the water insoluble formazan salt is produced. In comparison to these methods, the application of water soluble tetrazolium salts (.

WST-1, Takara Bio Inc., Japan) is found to be more sensitive in formation of water soluble cleaved formazan corresponding to the number of viable cells than formazan formed in MTT assay. To determine the effect of proliferation of MC3T3-E1 on the surface modified cover glass, the MC3T3-E1 cells at the density of 2.0 × 10^4^ cells/mL were seeded onto the Glass and the Glass-nHA, and incubated at 37 °C, 5% CO_2_ atmosphere in presence of DMEM medium. The extent of proliferation of MC3T3-E1 cells was monitored for the incubation time of 1 and 3 days. After incubation of MC3T3-E1 cells, 100 μL/well Premix WST-1 solution was added to each cell-seeded substrate, which was incubated using 1 mL DMEM/well. The final dilution ratio of WST-1 solution was kept in 1:10 and incubated for additional 2 h at 37 °C, 5% CO_2_ atmosphere in dark area. After the incubation, each 1 mL solution was divided into 10 in 96-well microtiter plates. 10% of WST-1 solution in no cell cultured medium was used as control. The absorbance was measured at 440 nm using 96 well kinetic microplate readers (ELx800, Bio-Tek Instruments, USA).

### Cell cytotoxicity

To evaluate the cell cytotoxicity on both the Glass and the Glass-nHA, the MC3T3-E1 cells were seeded on each substrate at the cell density of 2.0 × 10^4^ cells/mL. Cell-seeded substrates were incubated for 4 days at 37 °C, 5% CO_2_ atmosphere in presence of DMEM medium. After the culturing, the cells on substrate were stained with both calcein-AM and ethidium homodimer III dye solutions [15]. Staining solution was prepared by adding of 5uL calcein-AM (4 mM in anhydrous dimethyl sulfoxide) and 20uL of ethidium homodimer III (2 mM in DMSO/H_2_O) to 10 mL of PBS solution and mix thoroughly. After removing the DMEM from the culture well, the cell-adhered Glass and Glass-nHA were washed twice with PBS solution, and sufficient volume of staining solution was added to cover the cell monolayer. Subsequently, substrates were incubated for additional time in the dark area at the room temperature. After 30 min, staining solution was removed and the cells were washed three times with PBS and preserved in PBS solution until it is observed with confocal laser scanning microscope. Live and dead cells were simultaneously examined at the excitation wavelength of 494 nm for calcein-AM and 530 nm for ethidium homodimer III.

### Statistical analysis

All experimental data were collected in triplicates and presented as means ± standard deviations. The statistical analyses were performed using student’s two tailed test in conjunction with Scheffe’s test for multiple comparison statistics considering *p* < 0.05, *P* < 0.01, and *P* < 0.001 as statistically significant, very significant and extremely significant values, respectively, whereas *P* > 0.05 is treated as statistically insignificant value.

## Results

### Surface modification of glass and nHA

**Fig. 3:**
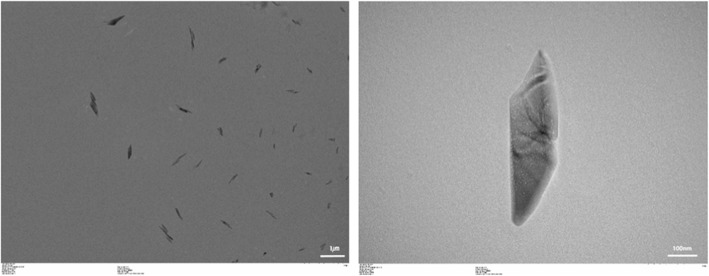
TEM micrographs of the nHAs prepared in this study

**Fig. 4:**
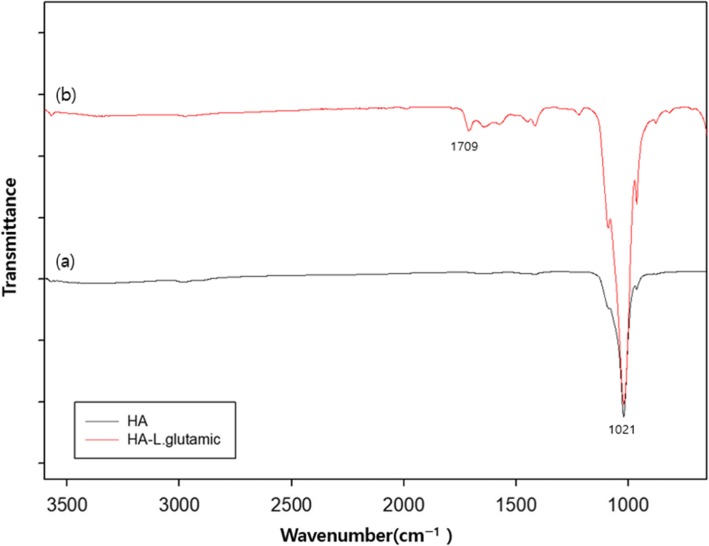
FT-IR spectra of the nHA (**a**) and the L-glutamic acid-grafted nHA (**b**)

To increase the surface functionalities of Glass, the Glass was firstly treated with piranha solution (1:3, H_2_O_2_:H_2_SO_4_), then treated with APTES solution to create a large number of amino groups on the surface of Glass (Fig. 1). In previous studies [14, 15], it was concluded that nHA was more suitable for dispersion and enhancement of osteogenic response to MC3T3-E1 cells in comparison to spherical hydroxyapatite. So, in this study, we tried to graft the nHA with the aim of enhancing the tissue compatibility of the Glass surface (Fig. 2). To graft the HA on the Glass, first of all, nHA was synthesized using procedure as reported in previous studies [14, 15]. The shape and size of nHA was confirmed by TEM micrographs. Hydroxyapatite prepared in this study has a rod shape and a size of 200 – 600 nm (Fig. 3). The nHA was used to graft onto the surface of Glass. Before adding the nHA to the Glass, it first reacted with L-glutamic acid. The grafting of L-glutamic acid on the nHA was confirmed by recording the FT-IR spectra on KBr pellets. The formation of L-glutamic acid-grafted nHA was confirmed by observing the presence of characteristic peaks of carboxyl groups at 1709 cm^− 1^ and phosphate groups of nHA at 1100 cm^− 1^ in FT-IR spectra (Fig. 4b).

### Grafting of nHA on the surface of glass

**Fig. 5:**
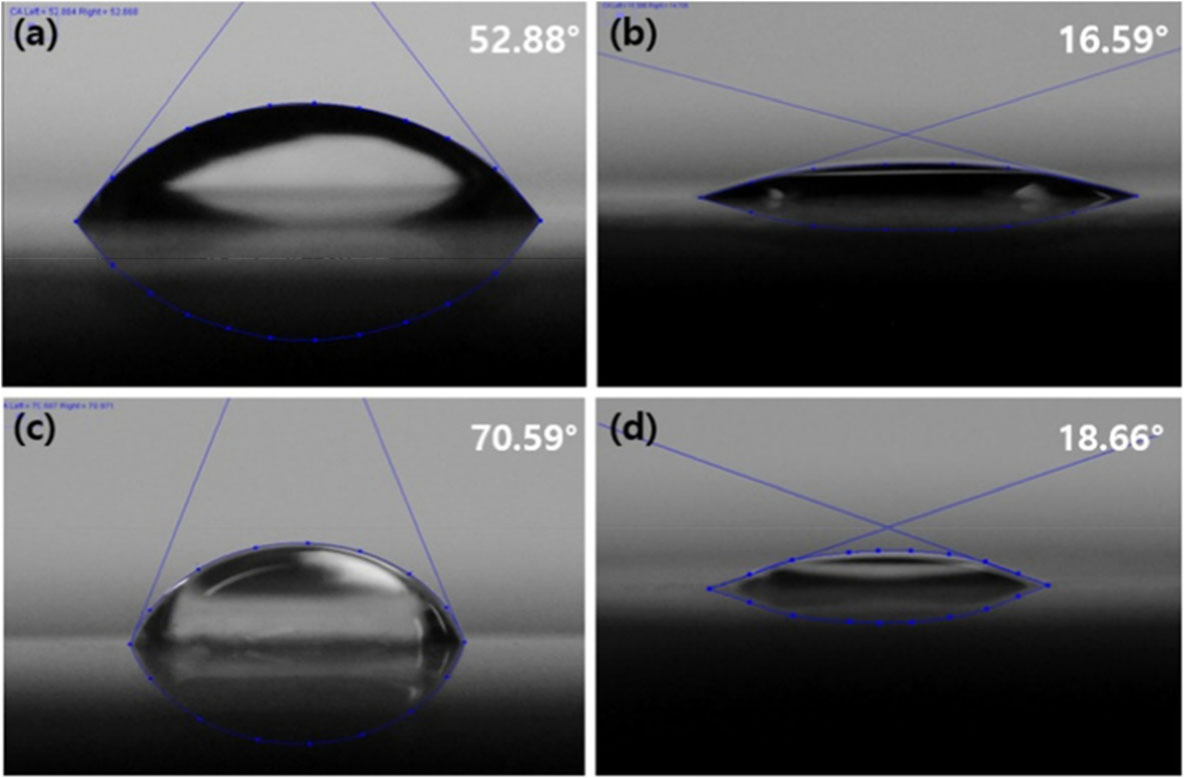
Water contact angle (θ) of the Glass (**a**), the piranha-treated Glass (**b**),the APTES-grafted Glass (**c**), and the Glass-nHA (**d**)

To prepare Glass-nHA, L-glutamic acid-grafted nHA was reacted with APTES-grafted Glass, as shown in Fig. 2. The presence of nHA on the Glass was confirmed by contact angle measurement, XPS and SEM. The water contact angles were measured to identify the wettability change of the cover glass depending on the surface modification. As shown in Fig. 5, contact angle decreased from 52^o^ to 16^o^ after cleaning the cover glass with piranha solution. Subsequently, the contact angle increased to 70^o^ with the introduction of APTES and then decreased to 18^o^ with the introduction of nHA.

**Fig. 6:**
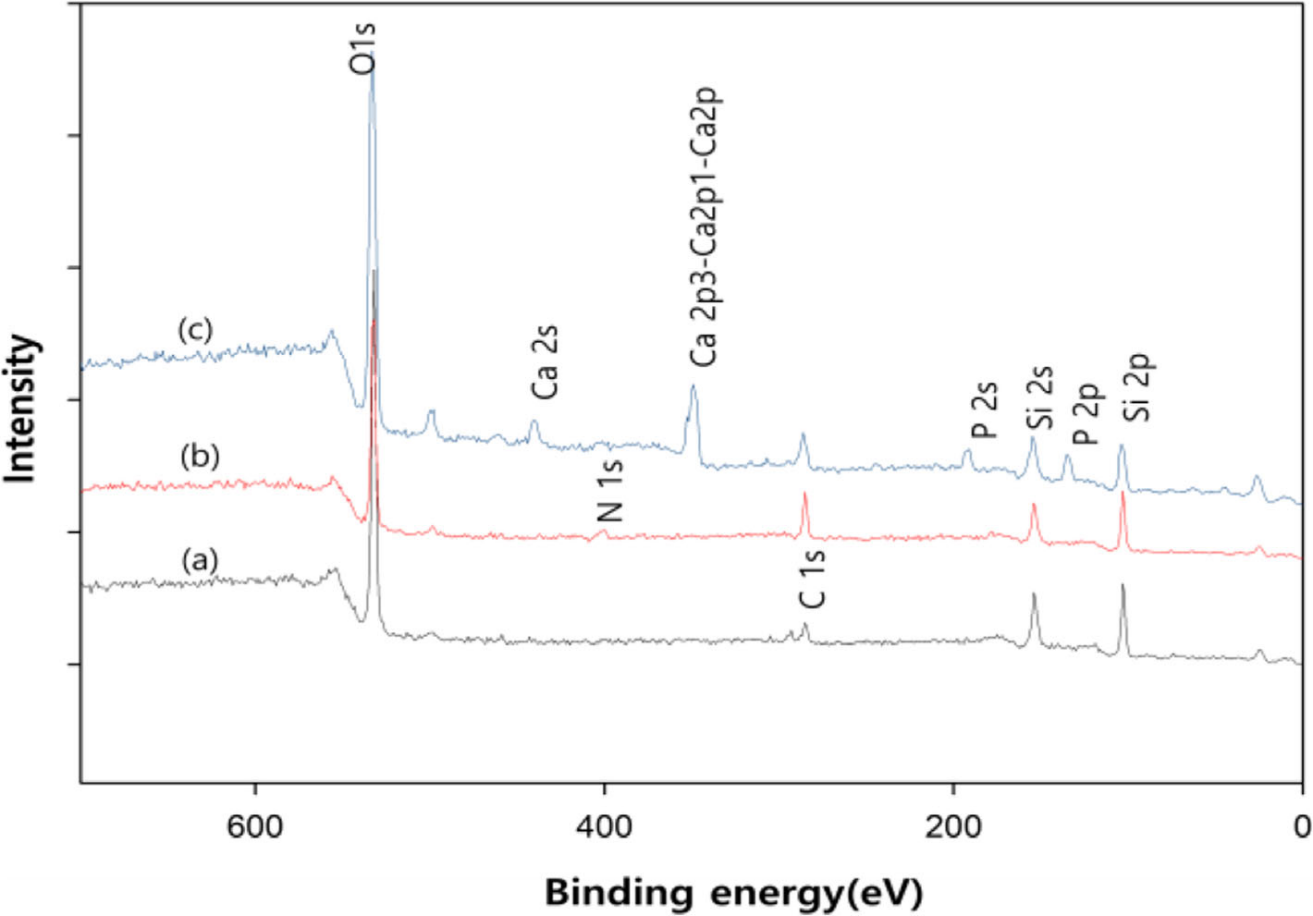
X-ray photoelectron spectra of the Glass (**a**), the APTES-grafted Glass (**b**) and the Glass-nHA (**c**)

XPS is a reliable method for surface element analysis. Thus an XPS analysis of surface-modified Glass was performed to ensure nHA was introduced on the Glass surface. The XPS results of the Glass and the APTES-grafted Glass have provided sufficient evidence for amino-functionalization of the Glass (Fig. 6). The characteristic binding energy peaks for Si2p and Si2s for silicon elements appeared at 104 eV and 154 eV, in all glasses (Fig. 6a, b). The Glass did not show any peak for N1 s while it was observed in the APTES-treated Glass at 400 eV, which clearly confirmed the presence of nitrogen due to amino functionalization of the Glass. The XPS quantitative data shown in Table 1 have clearly indicated that amino functionalized cover glass was having 2.94% of nitrogen while the Glass indicates 0% of nitrogen. These results have confirmed the presence of amino functional groups on APTES-grafted Glass. The presence of nHA could be confirmed by observing 347.9 and 133.2 eV based on Ca2_P_ and P2_p_, respectively (Fig. 6).

**Table 1:** XPS analysis of the Glass, the APTES-grafted Glass and the Glass-nHA

Sample	Atomic percentage of elements					
	Cls	Nls	Ols	Si2p	P2p	Ca2p
Glass	9.96	0.00	69.21	20.83	0.00	0.00
Glass-APTES	22.49	2.94	53.20	20.89	0.22	0.24
Glass-nHA	7.39	0.73	67.22	12.36	6.04	6.26

**Fig. 7:**
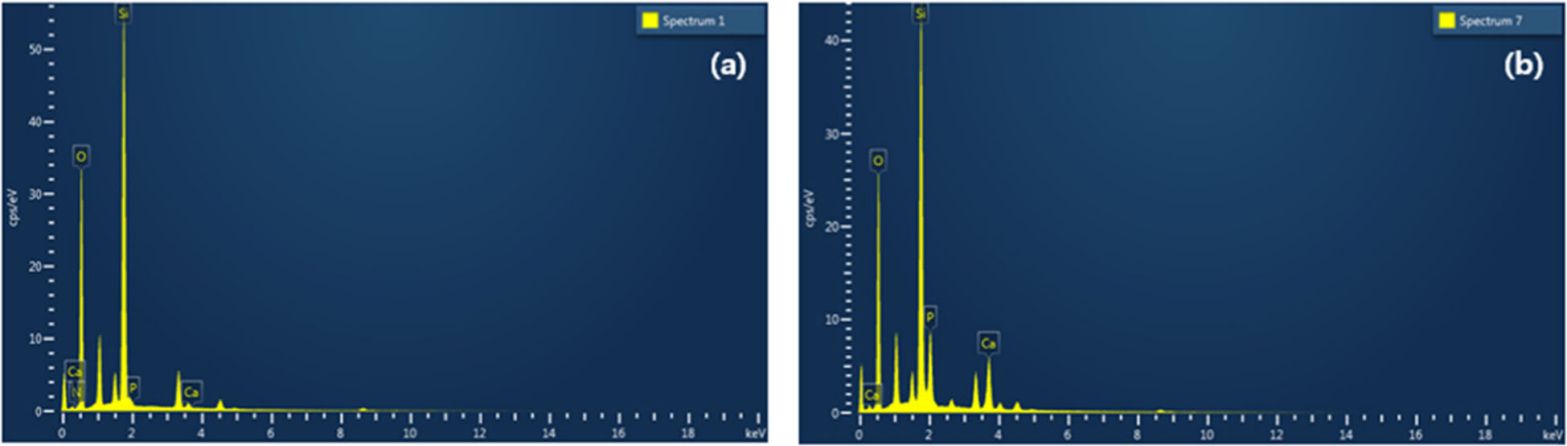
SEM-EDS elemental analysis on the Glass (**a**) and the Glass-nHA (**b**)

The EDS analysis for elements of the Glass (Fig. 7a, Table 2) and the Glass-nHA (Fig. 7b, Table 2) has further provided a concrete evidence for the grafting of nHA on the Glass. The weight and atomic percent for calcium (9.45 wt%) and phosphorus (8.04 wt%) of the Glass-nHA is found to be higher in comparison to the Glass (Table 2), which provided a quantitative evidence for the anchoring of L-glutamic acid-grafted nHA on the Glass.

**Table 2:** SEM-EDS quantitative data of the Glass and the Glass-nHA

Sample	Weight and (Atomic) percentage of elements				
	O	N	Si	P	Ca
Glass	55.32 (68.13)	0.75 (1.06)	43.92 (30.81)	0.0 (0.0)	0.0 (0.0)
Glass-nHA	51.00 (66.34)	0.75 (1.06)	31.51 (23.35)	8.04 (5.41)	9.45 (4.91)

Data in braces represents atomic weight percent

**Fig. 8:**
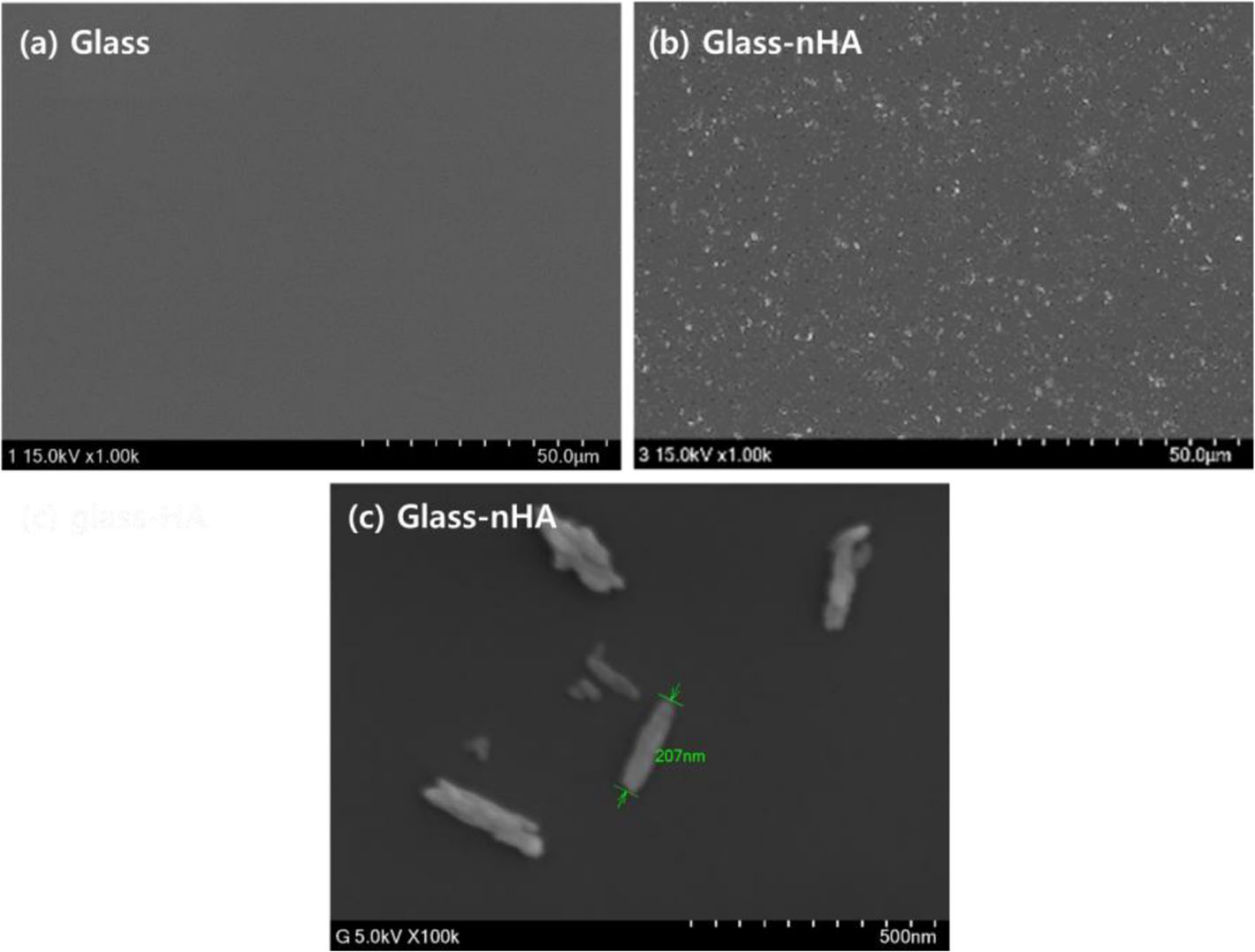
SEM surface images of the Glass (**a**) and the Glass-nHA (**b**, **c**)

Figure 8 shows SEM images of the Glass (a) and the Glass-nHA (b). As a result, the Glass showed a smooth surface (Fig. 8a). However, after nHA grafting, the nHAs were scattered over the Glass (Fig. 8b).

### Cell behaviors on glass-nHA

**Fig. 9:**
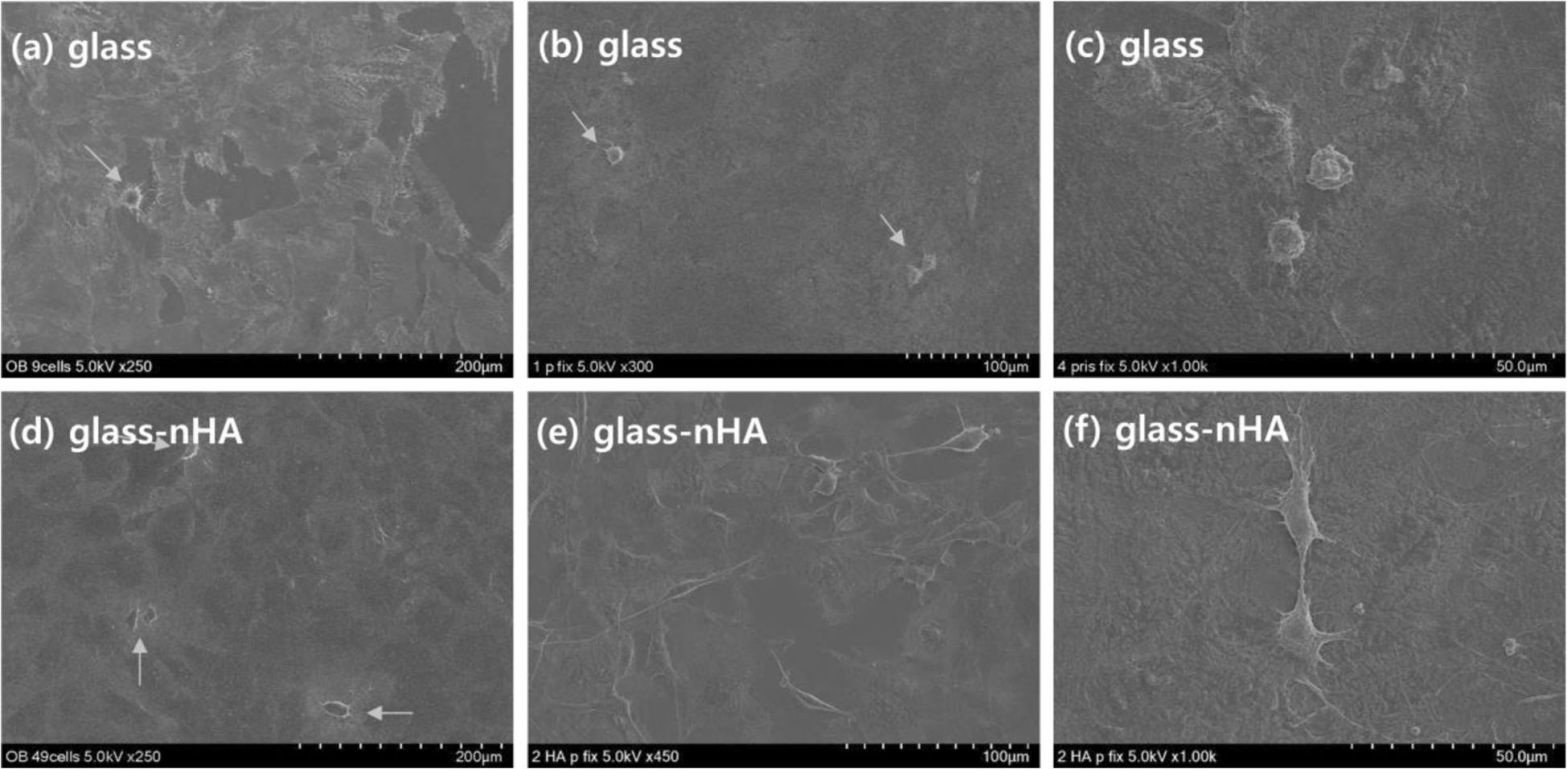
SEM images showing the adhesion of MC3T3E1 cells on the Glass (**a-c**) and the Glass-nHA (**d-f**). The cells were cultured at a density of 4 × 10^4^ cells/mL at 37 °C, 5% CO_2_ in humidified atmospheres for 1 day (a, d) and 2 days (**b, e**). (**c**), (**d**) are magnified images

**Fig. 10:**
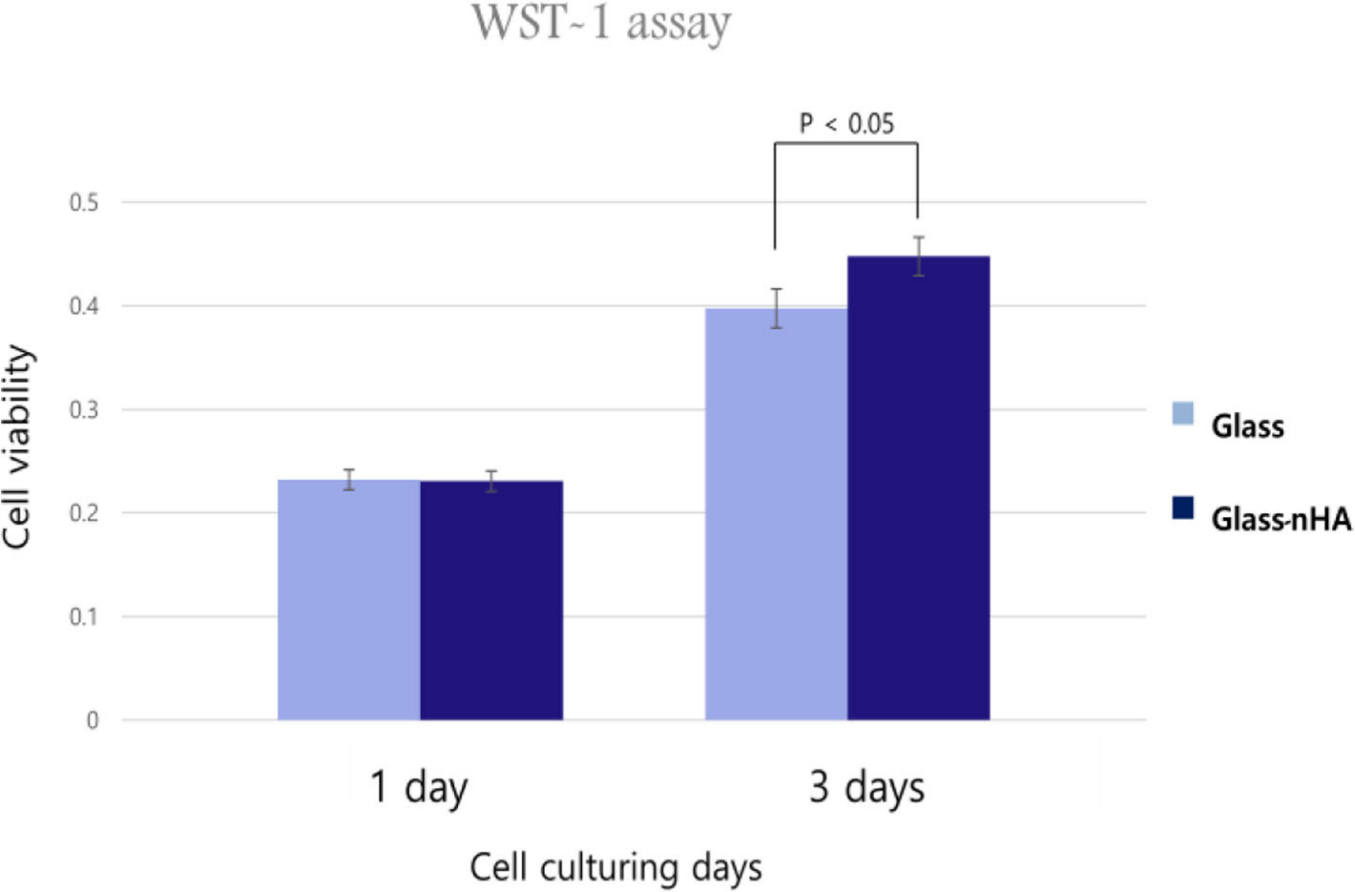
Proliferation of MC3T3 E1 cells which were cultured for 1 and 3 days on the Glass and the Glass-nHA

To evaluate the cell adhesion behavior, the MC3T3-E1 cells were seeded on the Glass and the Glass-nHA, and then incubated for 1 and 2 days in presence of DMEM medium. After incubation, cell morphologies were observed by SEM after fixing the cells with 2.5% glutaraldehyde solution followed by sputter-coating with platinum after critical point drier. After 1 day incubation, the cells did not spread enough on the pristine glass (Fig. 9a) and the glass-nHA (Fig. 9d). After 2 days incubation, the cells still did not spread on the glass (Fig. 9c), but they were spread well on the glass-nHA (Fig. 9e). The proliferation of MC3T3 E1 cells was evaluated for incubation period of 1 and 3 days to analyze the time dependent contribution of surface activity by recording the absorbance of water soluble formazan at 440 nm using ELISA 96well plate reader (Fig. 10). Cell growth rates after 1 day were not significiantly different in the Glass and the Glass-nHA, but 3 days later cells grew much better on the Glass-nHA. This is because the grafted nHA promoted the growth of osteoblasts.

**Fig. 11:**
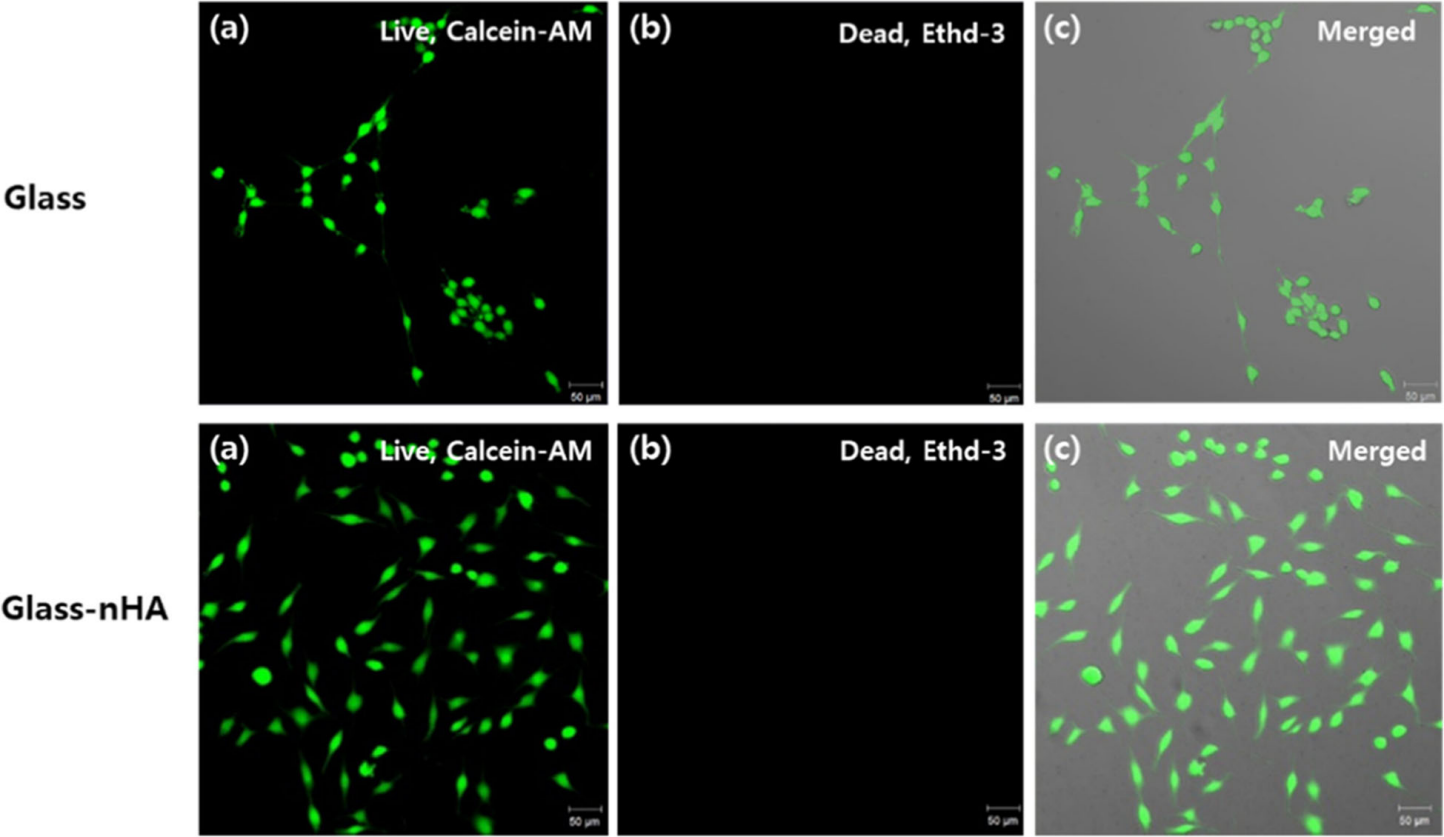
Live and dead assay of MC3T3 E1 cells which were cultured for 4 days on the Glass and the Glass-nHA. **a**; calcein AM, (**b**); ethidium homodimer III, (**c**); merged

To evaluate cytotoxicity of the Glass, MC3T3 E1 cells were seeded on each substrate with the cell density of 2.5 × 10^4^ cells/mL and incubated for 1–3 days. Toxic effect of the Glass surface was determined by staining cells with calcein-AM and ethidium homodimer III. As shown in Fig. 11, the green fluorescence indicates live cells, which could observe more cells on the Glass-nHA (Fig. 11b) in comparison to the Glass (Fig. 11a). The Glass and the Glass-nHA were shown to be non-toxic by all the stained cells being green and not red.

## Discussions

### Grafting of nHA to the glass

Glass ionomer cement (GIC) is a frequently used material as a filling agent when teeth are corroded. GIC consists of polymeric resins and inorganic microparticles, and the main ingredient of inorganic microparticles is silicate. Resin serves to enhance adhesion to the dentin or dental tissue, while inorganic particles maintain strength. We assumed that increasing the tissue compatibility of the surface of the silicate microparticle would eventually improve the tissue compatibility of the GIC. As a simple model of silicate among GICs, we selected silica Glass and examined its reactivity with nHA and tissue compatibility of Glass-nHA. To increase the reactivity of the Glass surface, APTES were introduced and L-glutamic acid was grafted to the surface of nHA. The presence of nHA on the Glass could be confirmed by tracing the change of water contact angle. The contact angle (θ) of the Glass decreased from 52^o^ to 16^o^ on treatment with piranha solution but increased to 70^o^ on amino-functionalization with APTES. The decrease in the value of contact angle to 16^o^ has clearly suggested that the surface of piranha-treated Glass was significantly populated with hydroxyl groups (Fig. 5b). The increase in the value of contact angle (70^o^) on treatment with APTES has clearly indicated that a large number of hydroxyl groups were changed to amino groups by APTES grafting. Residual hydroxyl groups participated in formation of hydrogen bonding with amino groups as reported in other studies [16, 17]. However, the contact angle again decreased to 18^o^ after nHA grafting. This is due to a number of hydroxyl groups in grafted nHA. We confirmed the grafting of nHA on the surface of Glass by measuring XPS. The XPS quantitative data shown in Table 1 have clearly indicated that the APTES-grafted Glass was having 2.94% of nitrogen while the pristine Glass indicates 0% of nitrogen. These results have confirmed the presence of amino functional groups on the APTES-grafted Glass. The presence of nHA could be confirmed by observing 347.9 and 133.2 eV based on Ca2_P_ and P2_p_, respectively (Fig. 6 and Fig. 1). Meanwhile, the identification of nHA that grafted to the Glass could also be done with scanning electron microscope. The Glass surface was uniform, while the Glass-nHA surface was very uneven. As shown in Fig. 8b,c, after nHA grafting, nanorod type of nHAs were scattered on the surface of the Glass.

### Osteoblast behaviors on glass-nHA

On comparing the SEM micrographs for cells adhesion on the Glass and the Glass-nHA (Fig. 9), it is clear that MC3T3-E1 cells were not spread on both the glass and the glass-nHA after 1 day incubation. However, after 2 days incubation, cells were well spread on the glass-nHA (Fig. 9f) while still not spread on the Glass (Fig. 9c). This is thought to be because surface’s hydroxyapatite stimulated the physiology of the osteoblasts. In addition, it is thought that the surface roughness [18] and wettability (18.66^o^) played a significant role in enhancing cell adhesion on the Glass-nHA. The evaluation of cell proliferation provides information about the biocompatibility, architecture and microenvironment that help in proliferation of adherent cells. It was clear that the Glass-nHA was more bioactive in supporting the proliferation of MC3T3 E1 cells in comparison to cells seeded on the Glass (Fig. 9). These results have clearly indicated that the presence of nHA on the Glass has helped in proliferation of MC3T3 E1 cells. On increasing the incubation time from 1 to 3 days, the extent of MC3T3 E1 cells proliferation on the Glass-nHA was significantly higher than that on the Glass (Fig. 10). In the live and dead assay using calcein-AM and ethidium homer dimer III, the stained cells were green, not red. The green fluorescence indicates live cells, which could observe more cells on the Glass-nHA (Fig. 11b) in comparison to the Glass (Fig. 11a). This has clearly indicated that the nHA has not only helped in controlling the surface bioactivity but also have not shown any toxicity of the cells. These data have indicated that the nHA has a potential to be used as a modifier of silicate microparticles in GIC for improving tissue compatibility.

## Conclusions

Amongst the ceramics, the hydroxyapatite is found to be highly osteogenic and its bioactivity is found to be dependent on its shape and size. These studies have indicated that the cell compatibility of Glass could be improved by the grafting of nHA on the Glass surface. The application of hydroxyapatites in the form of nanorod is more beneficial in controlling the bioactivity in comparison to spherical particles [19]. The grafting of L-glutamic acid-grafted nHA provided high surface area [14, 20] for interactions with tissue cells and to show high bioactivity for osteoblast cells. It is further indicated that the Glass chemically bonded with nHA is more supportive in controlling the tissue compatibility of the Glass due to its bioactivity of the nHA. The bioactivity of the Glass-nHA has provided a strong base for further application of inorganic biocomposites such as GIC etc.

## Data Availability

All data are available on journal portals in submitted manuscript. No other supporting files/data are needed along with this submission.

## Abbreviations

APTES: Aminopropyltriethoxysilane
GIC: Glass ionomer cement
Glass: Slide cover glass
Glass-nHA: nHA-grafted Glass
HA: Hydroxyapatite
nHA: Nanorod hydroxyapatite
